# How Plants Shut out Bacteria

**DOI:** 10.1371/journal.pbio.1001514

**Published:** 2013-03-19

**Authors:** Robin Meadows

**Affiliations:** Freelance Science Writer, Fairfield, California, United States of America

Unlike animals, which breathe through airways lined with pathogen-trapping defenses, plants get air through tiny pores in their leaves that all but invite bacteria to sneak in. How, then, do plants keep them out? They slam their pores, or stomata, shut. Stomata are flanked by guard cells that swell when triggered by bacteria, thus closing the pores. Besides being fascinating in its own right, this defense response has implications for human health—stomata have recently been shown to block some, but not all, types of the fecal bacteria that can contaminate leafy greens and other fresh produce, causing food poisoning outbreaks. In this issue of *PLOS Biology*, Montillet, Leonhardt, and colleagues show that guard cells contain an enzyme called lipoxygenase that makes stomata close in response to pathogens, overturning a previous theory that this process is regulated by the plant hormone abscisic acid.

Lipoxygenases have previously been linked to pathogen defenses in the plant *Arabidopsis thaliana*, and in the current work the researchers identified a lipoxygenase gene (LOX1) that is expressed in guard cells. To explore this gene's role in plant defenses, they sprayed LOX1-deficient *A. thaliana* with a strain of the bacterium *Pseudomonas syringae* that normally makes stomata close. However, they found that stomata barely closed in these mutant plants, showing that LOX1 is required for this defense against bacteria.

Bolstering this conclusion, Montillet and colleagues also found that substrates and products of the LOX1 enzyme make stomata close. Lipoxygenases turn fatty acids into fatty acid hydroperoxides (FAHs), and turn FAHs into reactive electrophile species (RES) oxylipins. The researchers found that stomata closed in response to two fatty acids that are LOX1 substrates, as well as in response to LOX1-produced FAHs and RES oxylipins.

Next, the researchers clarified the relative roles of LOX1 and abscisic acid (ABA), the plant hormone previously thought to mediate stomatal closure in this defense response. This set of experiments, which involved treating *A. thaliana* with a peptide from bacterial flagella that is known to activate plant defenses, yielded several lines of evidence that LOX1 and ABA activate guard cells via different pathways. For example, each pathway had two mitogen-activated protein kinases that were not in the other pathway. In addition, LOX1-produced RES oxylipins failed to activate OST1, a protein kinase in the ABA pathway. In contrast, RES oxylipins made stomata close in *A. thaliana* mutants that either lacked OST1 or could not synthesize ABA. Finally, stomata close within an hour of the flagellar peptide treatment, but ABA marker genes were not expressed, suggesting that ABA was not produced during this time period.

Moreover, Montillet and colleagues discovered that only the LOX pathway includes another plant hormone called salicylic acid, which is required for stomatal closure in response to bacteria. They found that the RES oxylipins produced by LOX1 failed to activate guard cells in *A. thaliana* mutants that lacked salicylic acid. In contrast, ABA still activated guard cells in these salicylic acid–deficient mutants. Besides suggesting that ABA and LOX1 are in different pathways, this work suggests that the LOX1 pathway makes plants synthesize salicylic acid, and that this is a key step in activating guard cells to shut out bacteria.

So what does trigger the ABA pathway? Guard cells also close in response to environmental conditions such as light and humidity, suggesting that ABA mediates stomatal closure in response to such abiotic factors rather than to biological ones.

While the researchers conclude that the ABA and LOX1 pathways are distinct and have different triggers, they also stress that both are required for stomata to close in response to bacteria. This is because regardless of whether the trigger is biotic or abiotic, ABA is the main regulator of the membrane proteins that control osmotic pressure in guard cells, which is what determines whether stomata are open or shut. Notably, an ion channel called SLAC1 is required for both biotic and abiotic stomatal closure, suggesting that both the ABA and LOX1 pathways act on this membrane protein.

These findings extend previous work showing that stomata are key players in the defense response of plants, and significantly advance our understanding of stomatal closure. This could ultimately help us protect people from food plants that can carry human pathogens. In addition, because stomata regulate water loss, teasing apart the abiotic and biotic pathways of stomatal closure could help us breed plants better suited to harsh environmental conditions.

**Montillet J-L, Leonhardt N, Mondy S, Tranchimand S, Rumeau D, et al. (2013) An Abscisic Acid-Independent Oxylipin Pathway Controls Stomatal Closure and Immune Defense in *Arabidopsis*. doi:10.1371/journal.pbio.1001513**

